# Thiamine Status in Humans and Content of Phosphorylated Thiamine Derivatives in Biopsies and Cultured Cells

**DOI:** 10.1371/journal.pone.0013616

**Published:** 2010-10-25

**Authors:** Marjorie Gangolf, Jan Czerniecki, Marc Radermecker, Olivier Detry, Michelle Nisolle, Caroline Jouan, Didier Martin, Frédéric Chantraine, Bernard Lakaye, Pierre Wins, Thierry Grisar, Lucien Bettendorff

**Affiliations:** 1 GIGA-Neurosciences, University of Liège, Liège, Belgium; 2 Department of Cardiovascular Surgery, University Hospital, University of Liège, Liège, Belgium; 3 Department of Abdominal Surgery and Transplantation, University Hospital, University of Liège, Liège, Belgium; 4 Department of Gynecology-Obstetrics, Citadelle Hospital, University of Liège, Liège Belgium; 5 Department of Neurosurgery, University Hospital, University of Liège, Liège, Belgium; Universidad Peruana Cayetano Heredia, Peru

## Abstract

**Background:**

Thiamine (vitamin B1) is an essential molecule for all life forms because thiamine diphosphate (ThDP) is an indispensable cofactor for oxidative energy metabolism. The less abundant thiamine monophosphate (ThMP), thiamine triphosphate (ThTP) and adenosine thiamine triphosphate (AThTP), present in many organisms, may have still unidentified physiological functions. Diseases linked to thiamine deficiency (polyneuritis, Wernicke-Korsakoff syndrome) remain frequent among alcohol abusers and other risk populations. This is the first comprehensive study on the distribution of thiamine derivatives in human biopsies, body fluids and cell lines.

**Methodology and Principal Findings:**

Thiamine derivatives were determined by HPLC. In human tissues, the total thiamine content is lower than in other animal species. ThDP is the major thiamine compound and tissue levels decrease at high age. In semen, ThDP content correlates with the concentration of spermatozoa but not with their motility. The proportion of ThTP is higher in humans than in rodents, probably because of a lower 25-kDa ThTPase activity. The expression and activity of this enzyme seems to correlate with the degree of cell differentiation. ThTP was present in nearly all brain and muscle samples and in ∼60% of other tissue samples, in particular fetal tissue and cultured cells. A low ([ThTP]+[ThMP])/([Thiamine]+[ThMP]) ratio was found in cardiovascular tissues of patients with cardiac insufficiency. AThTP was detected only sporadically in adult tissues but was found more consistently in fetal tissues and cell lines.

**Conclusions and Significance:**

The high sensitivity of humans to thiamine deficiency is probably linked to low circulating thiamine concentrations and low ThDP tissue contents. ThTP levels are relatively high in many human tissues, as a result of low expression of the 25-kDa ThTPase. Another novel finding is the presence of ThTP and AThTP in poorly differentiated fast-growing cells, suggesting a hitherto unsuspected link between these compounds and cell division or differentiation.

## Introduction

**Figure 1:**
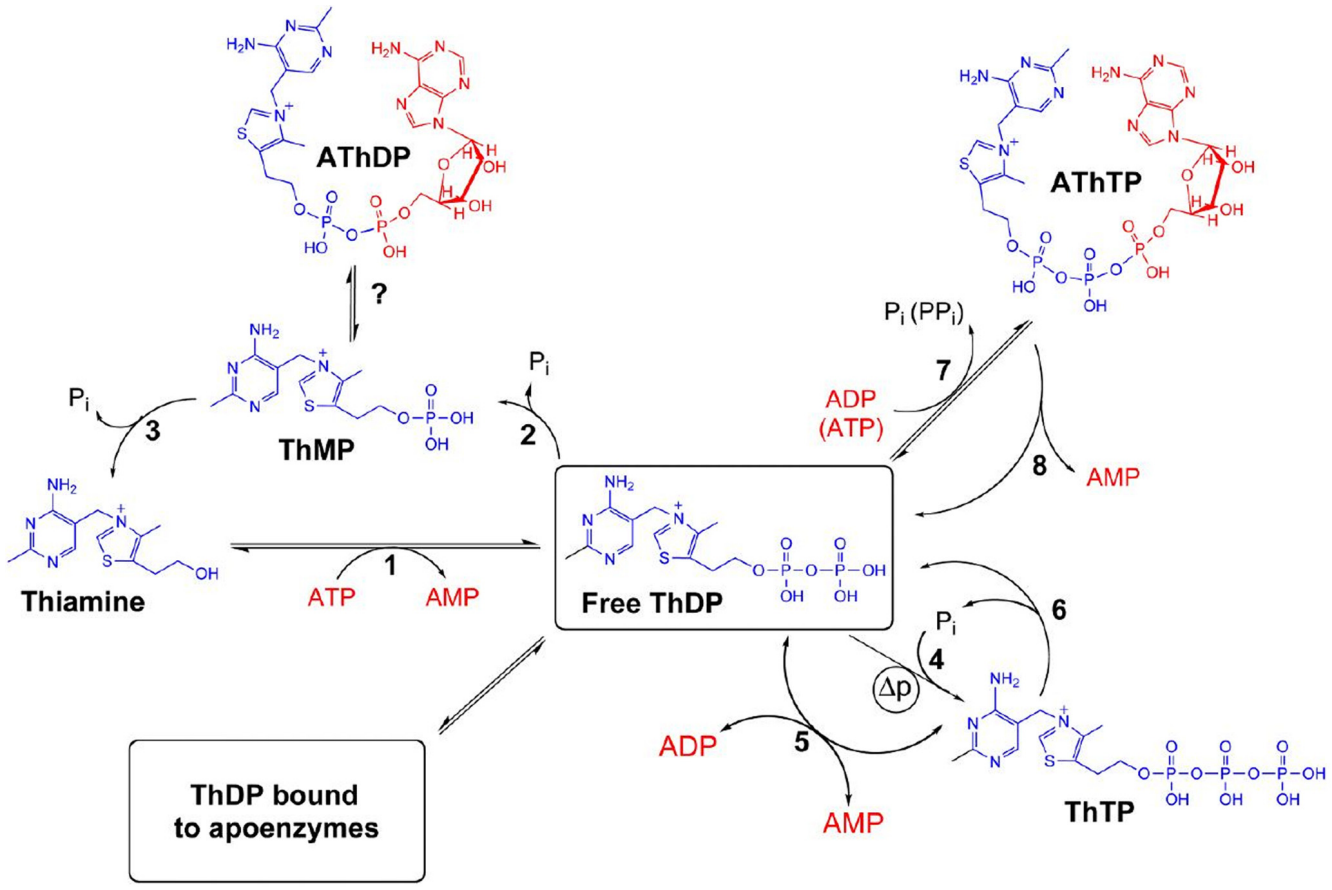
Interconversion of thiamine derivatives in a model human cell. 1, cytosolic thiamine diphosphokinase (TPK); 2, cytosolic thiamine diphosphatase (also hydrolyzes nucleoside diphosphates); 3, thiamine monophosphatase (hypothetical); 4, mitochondrial membrane-associated ThTP synthase; 5, cytosolic adenylate kinase; 6, cytosolic 25-kDa ThTPase; 7, cytosolic ThDP adenylyl transferase; 8, AThTP hydrolase (hypothetical). The mechanisms of AThDP synthesis and degradation are unknown. Δp, transmembrane H^+^ gradient. Updated from [21].

Thiamine is an essential molecule for all life forms. In animal cells, thiamine is phosphorylated to ThDP by a specific enzyme, thiamine pyro(di)phosphokinase (TPK, EC 2.7.6.2) (Fig. 1). As ThDP is a cofactor for transketolase and pyruvate and 2-oxoglutarate dehydrogenase complexes required for the oxidative degradation of sugars and mitochondrial synthesis of ATP, thiamine deficiency results in acute energy failure. In contrast to microorganisms and plants, which are able to synthesize thiamine *de novo*, animals require an exogenous source. Thiamine deficiency in humans causes beriberi, a peripheral neuropathy and, mostly in alcohol-misusing patients, Wernicke-Korsakoff syndrome [1], [2]. Based on postmortem analysis, the prevalence of the latter is 1–2% of the general population and 12–14% of the misusing population [3], making it the third commonest cause of dementia, after Alzheimer's disease and vascular dementias [2]. Marginal thiamine deficiency is probably more common than thought in the elderly [4], [5], [6], [7], in risk groups such as HIV-positive patients [8], [9], [10], in patients with fast-growing hematologic malignant tumors [11], after chronic liver failure [12] or following gastrectomy [13]. Accidental thiamine deficiency was also recently documented by the 2003 outbreak of encephalopathy in infants in Israel, caused by a defective soy-based formula [14].

Thiamine deficiency or defects in thiamine metabolism were also reported in other human pathologies. While one study reported normal total blood thiamine levels in patients with Alzheimer's dementia [15], others showed decreased levels in plasma or erythrocytes [16], [17]. ThDP levels are decreased in postmortem brains of patients with Alzheimer's disease [18], [19] and frontal lobe degeneration of the non-Alzheimer's type [20].

Thiamine deficiency induces neuronal loss and cardiac failure. The brain and the heart have an absolute requirement for oxidative metabolism, and it is generally thought that the clinical symptoms observed during thiamine deficiency result from decreased tissular ThDP levels. However, in addition to the well-known cofactor ThDP, other thiamine derivatives might also play physiological roles [21] (Fig. 1). ThTP exists in animal tissues in variable amounts [22]. ThTP is able to phosphorylate proteins [23] and to activate large conductance anion channels in excised patches of mouse neuroblastoma cells [24], but the physiological significance of these results remains to be proven.

The mechanism of ThTP synthesis has long been controversial. It now appears that there are at least two different mechanisms. Kawasaki and coworkers showed that adenylate kinase 1 (AK1) is able to synthesize ThTP according to the reaction ThDP + ADP 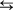 ThTP + AMP [25], [26]. This reaction may lead to a significant synthesis of ThTP in skeletal muscle where this enzyme is particularly abundant. We have shown that synthesis of ThTP is a feature of all known AKs and that this reaction is always 10^6^–10^7^ times slower than ATP synthesis according to the reaction 2 ADP 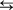 ATP + AMP [27]. In brain however, ThTP is synthesized in mitochondria by a chemiosmotic mechanism, according to the reaction ThDP + P_i_ 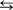 ThTP coupled to the respiratory chain [28].

The enzymatic hydrolysis of ThTP has been reported in many tissues. Mammalian tissues contain a membrane-associated ThTPase [29], [30], [31] and a soluble cytosolic ThTPase [32]. The membrane-associated ThTPase has not been characterized at the molecular level. The soluble ThTPase (EC 3.6.1.28) is a 25-kDa protein belonging to the CYTH superfamily [33] or triphosphate tunnel metalloenzymes [34] and its three-dimensional structure is known [35]. This ThTPase is expressed in virtually all mammalian tissues [22], [36], [37] and appears to be mainly neuronal in brain [38]. Orthologs of the 25-kDa ThTPase are present in all animal species, except possibly in birds [22].

Recently, we discovered the first thiamine adenine nucleotide, adenosine thiamine triphosphate (AThTP), which accumulates in *E. coli* during carbon starvation or collapse of the membrane H^+^ gradient [39], [40]. AThTP is also present in mammalian tissues. In *E. coli* it can be synthesized by a ThDP adenylyl transferase, present in the cytosol [41], according to the reaction ThDP + ADP 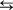 AThTP + P_i_. In carbon-starved *E. coli*, it rapidly disappears after addition of glucose [39], suggesting the presence of an AThTP-hydrolyzing enzyme. Furthermore, another adenine thiamine nucleotide, adenosine thiamine diphosphate (AThDP), exists, at least in mouse and quail liver [42], but we no information concerning its synthesis or degradation.

We only begin to understand the metabolism of thiamine derivatives and in particular ThTP and AThTP. There are only a few studies on the distribution of these compounds in animal tissues [22], [42] and only two (from the same group) in humans showing decreased ThTP levels in the postmortem brains of patients with subacute necrotizing encephalomyelopathy (Leigh's disease) [43], [44]. However, the compound measured in the latter study may not have been authentic ThTP [45]. Indeed, ThTP measurements were unreliable before the development of HPLC techniques and we were unable to detect ThTP in human postmortem brains [18], [46], probably because of hydrolysis during the postmortem delay.

Therefore, it is important to obtain information on the thiamine status in humans, which can only be done reliably using fresh tissue samples, especially in the case of ThTP and AThTP, which are subject to rapid metabolic degradation. This is the first study on the content of thiamine derivatives in various biopsied human tissue samples and some human cultured cells. We also checked the expression of the 25-kDa ThTPase by determination of its enzyme activity, immunoblotting and RT-PCR. The results obtained allow us to draw several interesting conclusions concerning the distribution and the abundance of thiamine compounds in humans.

## Results

### Biological stability and turnover of thiamine derivatives in biological samples

A general problem associated with the detection of metabolites in tissues is related to their biological stability. ATP, for instance has a very high turnover with a half-life <1 s. Thus, even the short delay between the sacrifice of the animal and the freezing of the target organ is sufficient for most of the ATP to be hydrolyzed [47], [48]. Reliable estimations of cellular ATP concentrations are therefore difficult to obtain. The turnover of thiamine derivatives is much slower, so that this issue is less important. We previously showed that in cultured mouse neuro-2a cells, the turnover time of ThDP is ∼17 h at 37°C [49]. In a previous study on human brain samples, we did not find a significant effect of the postmortem delay (3–27 h, n = 59) on ThDP levels [46], suggesting that reliable estimations of ThDP content can be obtained on human postmortem samples. However, it should be kept in mind that a hydrolysis of only few percent of ThDP during the postmortem delay would often go unnoticed but would lead to an important increase in the comparatively minor ThMP, the product of hydrolysis of ThDP. In the case of ThTP, the turnover time is ∼1 h in neuro-2a cells which is slow enough for no significant hydrolysis to occur during a normal sampling procedure. However, a strong underestimation would be obtained for longer postmortem delays. Indeed, in previous studies, we were unable to detect any ThTP in human brain postmortem samples [18], [46]. Therefore, a reliable estimation of ThTP content can only be obtained from human biopsies. No data are available concerning the biological stability of adenylated thiamine compounds in animal tissues, but in carbon-starved *E. coli*, AThTP disappears within a few minutes after addition of glucose [39].

### Thiamine compounds in human blood and cerebrospinal fluid (CSF)

Thiamine phosphate esters are hydrolyzed in the intestinal lumen by various phosphatases (mainly the alkaline phosphatase associated with brush-border membranes) and thiamine is taken up through the intestinal mucosal membrane by a specific saturable transporter (*K*_m_ = 4.4 µM) through an energy-dependent process [50], [51]. The vitamin is transported into erythrocytes by another saturable, high-affinity transporter (*K*_m_ = 0.11 µM) and a non-saturable component [52] that is probably not relevant at physiological plasma concentrations. Indeed, the total (free + protein-bound) thiamine concentration in human plasma is very low (10–20 nM), while it reaches over 100 nM in rodent plasma, which in addition also contains high amounts of ThMP [53]. Inside the erythrocytes, thiamine is pyrophosphorylated to ThDP by TPK according to the reaction Thiamine + ATP 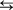 ThDP + AMP. Though the equilibrium constant for this reaction is not in favor of ThDP formation [54], the steady-state ThDP concentration in erythrocytes is much higher than that of free thiamine. It is known that ThDP binds to transketolase with high affinity, but the molar concentration of the enzyme is probably too low to explain the shift of the equilibrium towards ThDP synthesis. Another explanation is that the AMP formed is converted to ADP by AK1, which is abundant in human red blood cells [54]. This would explain that the free ThDP can accumulate to some extent in the cytoplasm and is trapped in the cell (as it cannot cross the membrane). In human erythrocytes, up to 10% of the free ThDP can be converted to ThTP, a reaction catalyzed (albeit very slowly) by AK1 according to the reaction ThDP + ADP 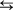 ThTP + AMP [54].

Many different methods for the determination of thiamine derivatives in human whole blood, erythrocytes, serum or plasma have been described and it is not the aim of the present work to review all these data, which has been done previously [55], [56]. Here, we essentially wanted to investigate the possible presence of ThTP and AThTP in these fluids.

In agreement with previous results [57], [58], [59], [60], whole human blood contained thiamine, ThMP, and ThDP (Table 1). ThTP accounted for nearly 10% of total thiamine, which is more than in most human cells, while ThMP and free thiamine were less abundant. We detected no significant amounts of AThDP or AThTP in human blood. Plasma contained low amounts of thiamine and ThMP, but neither ThDP nor ThTP. Similar concentrations of free thiamine (10–20 nmol/L) in human plasma or serum have been reported by us [61] and other workers [59], [61], [62]. In human blood, total thiamine concentrations are nearly an order of magnitude lower than in blood of rodents and other animals [53], [63].

**Table 1:** Distribution of thiamine derivatives in human whole blood, plasma and CSF.

(nmol/L)	Thiamine	ThMP	ThDP	ThTP	AThTP
Whole blood (7)	4±3	10±4	138±33	13±4	n. d.
Plasma (3)	11±3	5±2	n. d.	n. d.	n. d.
CSF (3)	19±5	30±5	n. d.	n. d.	n. d.

The blood was from 7 healthy volunteers aged from 25 to 49 years. Aliquots of three samples were centrifuged to obtain a plasma preparation. CSF was from three patients with no known neurological disorder. The results are expressed as means ± SD. The numbers in parentheses indicate the number of patients.

n. d., not detected.

In human CSF, we only found thiamine and ThMP, in agreement with Rindi and coworkers [64]. ThMP is probably transported through the choroid plexus by the reduced folate transporter (SLC19A1), that is very abundant in the apical (CSF) side of the choroid plexus [65] and able to transport ThMP (but not thiamine) in addition to reduced folate [66].

### Thiamine compounds in various tissues including the cardiovascular system

We determined thiamine compounds in biopsies from various human tissues, including colon, lung, thymus, skin, skeletal muscle and various elements of the cardiovascular system from 33 patients (Table 2). In some cases, several kinds of anatomically different samples from similar tissues (veins, arteries or valves) were pooled for statistical reasons, as there were no obvious differences between them. The 33 patients were aged between 37 and 82 years. At first, it appeared that the content of thiamine compounds was highly variable from one patient to another, as evidenced by the high coefficient of variation (C_v_), often >0.7. ThMP and ThDP were found in all patients. Free thiamine and ThTP were found in only half of the samples. Skeletal muscle always contained measurable amounts of ThTP, except for one patient aged 82 with also very low ThDP levels. The presence of ThTP (mostly cytosolic) in skeletal muscle is probably related to the high content of AK1. As mentioned above, erythrocytes contain relatively high amounts of ThTP for probably the same reason.

**Table 2:** Distribution of thiamine derivatives in human biopsies.

(pmol/mg of protein)	Thiamine	ThMP	ThDP	ThTP	AThTP
Colon (2)	0.07±0.06	2.1±2.4	30±22	0.3±0.2	n. d.
Lung (7)	2.2.±1.1	2.0±0.9	30±12	0.49 [1]	0.43 [1]
Kidney (1)	3.5	80	33	0.19	n. d.
Thymus (6)	0.23±0.16 [2]	1.1±0.4	7.1±0.9	1.1 [1]	0.81 [1]
Skin (3)	2.1±0.6	3.6±0.8	47±12	0.44±0.09	0.13±0.05 [2]
Adipose tissue (11)	3±4 [4]	3±3	27±23	2±2 [5]	0.8±0.4 [2]
Skeletal muscle (11)	0.6±0.7 [7]	0.7±0.4	17±12	1±1 [10] 4	1.5±1.4 [2]
Heart auricle (5)	0.63 [1]	1.4±0.7	66±41	0.4±0.5 [4]	n. d.
Pericardium (6)	1.3±0.6 [3]	0.8±0.5	4.8±2.3	0.25 [1]	n. d.
Veins^1^ (10)	1.4±1.0 [6]	1.7±1.4	9±6	3±4 [6]	1.4±1.4 [3]
Arteries^2^ (6)	1.0±0.8 [4]	2.4±2.0	10±7	0.8±0.3 [4]	7±9 [2]
Valves^3^ (5)	2.6±3.6 [2]	0.9±0.5	4.7±2.2	1.8±1.5 [2]	1.3±1.2 [2]

Among the 33 patients, 29 were hospitalized because of cardiovascular problems. The results are expressed as means ± SD. The numbers in parentheses indicate the number of patients. The numbers in brackets indicate the number of samples in which the compound was observed, if different from the number of patients.

n.d., not detected.

1 great sapheneous vein, mammary vein,

2 mammary artery, radial artery, coronary artery.

3 mitral valve, aortic valve.

4 One patient, of 82 years, had no measurable ThTP in the skeletal muscle sample. This sample also had a low ThDP content (5 pmol/mg of protein).

In most tissues investigated, the cofactor ThDP was the most abundant thiamine compound, with the highest content in heart auricles and skin, followed by kidney, lung, colon, adipose tissue, skeletal muscle and vascular samples. We tested whether there was a correlation between the age of the patients and the content of thiamine derivatives. Indeed, using linear regression analysis, we found a significant negative correlation between age and ThDP levels. The ThDP content decreased with the age of the patients in skeletal muscle [*F*(1,9) = 6.701, p = 0.0293, R^2^ = 0.43] and lung [*F*(1,5) = 14,730, p = 0,0121, R^2^ = 0.75], for which we had the most complete series of samples. No significant correlation was found for ThTP [*F*(1,8) = 0.6348, p = 0.45, R^2^ = 0.074] or ThMP [*F*(1,9) = 1.547, p = 0.25, R^2^ = 0.15] in skeletal muscle, but a significant negative correlation between ThMP and age [*F*(1,5) = 11.853, p = 0.0184, R^2^ = 0.70] was observed in lung. A significant negative correlation was also found between total thiamine content and age in skeletal muscle [*F*(1,9) = 9.408, p = 0.0134, R^2^ = 0.51] and lung [*F*(1,5) = 17.6073, p = 0.0085, R^2^ = 0.78]. In a previous study limited to human postmortem brains, we already observed a tendency for decreased ThDP and total thiamine levels with age, and in particular in the highest age group (≥77 years) [46], though TPK activities were normal.

In veins and arteries, ThDP levels were relatively low, while ThTP levels were proportionally high, accounting in two patients for about 15%–16% of total thiamine. This seems to be a characteristic of human tissues, as in aorta and vena cava from rats, ThTP represented only respectively 0.6% (0.27 pmol/mg of protein, n = 3) and 0.3% (0.23 pmol/mg of protein, n = 3) of total thiamine (not shown).

It is interesting to note that, in some patients, ThTP was below detection limit in all tissues, except skeletal muscle. In these patients, free thiamine was present, sometimes in high amounts. Therefore, we separated the patients in two categories: those with a “positive” thiamine phosphorylation shift (toward ThTP) and those with a “negative” thiamine phosphorylation shift (toward thiamine). We calculated the ([ThTP] + [ThMP])/([Thiamine] + [ThMP]) ratio for each patient by taking the mean of all tissues from the same patient. We have previously shown that there are at least two ThDP pools, a larger low-turnover cofactor pool and a smaller high-turnover pool, precursor of ThTP and ThMP [49], [67]. Therefore, as we do not know the size of the free high-turnover ThDP pool, this derivative was omitted from the equation. We called this ratio the “thiamine phosphorylation ratio” (TPR). 20 patients (61%) had a TPR ≥1, while 11 (39%) had a TPR <1. We did not find a significant correlation between TPR and total thiamine content in our samples [*F*(1,28) = 0.0059, p = 0.94, R^2^ <0.0001]. Also, the TPR did not correlate with the age of the patients [*F*(1,28) = 0,09719, p = 0,7575, R^2^ = 0,003459].

From the retrospective analysis of the clinical charts, we did not find arguments for a relation between underlying neoplastic disease and a low TPR. However, among the patients with low TPR all but one had a significant degree of heart failure based on clinical assessment (New York Heart Association Functional Classification) and echocardiographic data. The heart failure syndrome is characterized by a diffuse neuro-hormonal activation and metabolic shifts [68], [69]. Therefore, this possible and plausible correlation warrants further study and is currently under prospective investigation in our institution.

As for AThTP, it was detected in only 15 samples from 10 patients (out of 33). AThTP was present in 35% of the samples containing AThTP, but in only 3% of the samples devoid of ThTP. This difference is highly significant (p = 0.0009), suggesting that the appearance of AThTP is linked to the presence of ThTP. This is in contrast to what is observed in bacteria where ThTP and AThTP do not generally accumulate together [39].

### Thiamine derivatives in normal and ischemic human liver

We thought that ischemia might affect the distribution of thiamine derivatives in liver tissue. Indeed, it is known that thiamine protects against hypoxia-induced cell death in cultured neonatal rat cardiomyocytes [70] and accelerates the healing of diabetic ischemic limbs in mice [71]. This may be one of the reasons why thiamine supplementation improves renal transplantation outcome in humans [72]. However, we did not observe any effect of ischemia on the levels of thiamine compounds in liver (Table 3). Neither AThDP nor AThTP were found in human liver, in contrast to mouse [42] or rat [39] liver. Human liver contained significant amounts of ThTP (>1% of total thiamine), and ∼73% of ThTP was found in the supernatant after centrifugation of the liver homogenate at 100 000× g. This may be a difference with brain or cardiac tissue, where a large part of ThTP is mitochondrial [28] and might therefore be more sensitive to ischemia.

**Table 3:** Distribution of thiamine derivatives in normal and ischemic human liver.

(pmol/mg of protein)	Thiamine	ThMP	ThDP	ThTP	AThTP
Normal (3)	0.26±0.07	3±2	45±29	1.7±1.8^a^	n. d.
Ischemic (3)	0.9±0.8	4±3	48±15	0.2±0.2	n. d.

The results are expressed as means ± SD. The numbers in parentheses indicate the number of patients.

a One patient had a very high ThTP content (3.8 pmol/mg, or 12% of total thiamine).

n.d., not detected.

### Thiamine compounds and 25-kDa ThTPase in gynecological and embryonic tissues

As the fetus continuously grows, it acts as a thiamine sink, explaining a 10 to 1 ratio of the thiamine concentrations in the maternal versus venous cord plasma [73]. ThDP was the major compound observed in human adult gynecological specimen and in embryonic tissues (Table 4). A high amount of ThMP was also found in several samples such as fibroma, ligament, serosa and endometrium. It could be argued that this high proportion of ThMP is the result of a hydrolysis of ThDP after the sample was taken. However, we took care, first, to freeze the samples as quickly as possible and the delay was never longer than a few minutes (generally less than 2 minutes). Secondly, we previously showed that, at least in the human brain, a postmortem delay of several hours does not significantly change the relative thiamine, ThMP or ThDP levels [18], [46], suggesting that ThDP hydrolysis is slow. Indeed, though some nucleoside diphosphatases are able to hydrolyze ThDP [74], [75], no specific thiamine diphosphatase has yet been characterized. This does however not exclude that in some tissues unspecific phosphatases might be responsible for a rapid hydrolysis of ThDP. This is in contrast to ThTP, which rapidly disappears during the postmortem delay, probably because of the action of the specific 25-kDa ThTPase or mitochondrial phosphohydrolases [28].

**Table 4:** Distribution of thiamine derivatives in human embryonic tissues and adult gynecological specimen.

(pmol/mg of protein)	Thiamine	ThMP	ThDP	ThTP	AThTP
Placenta (4)	1.7±0.5	0.9±0.8	15±10	0.12±0.07	0.9±0.6
Umbilical cord (2)	3.1±0.9	0.8±0.4	5±4	0.13 [1]	0.17 [1]
Trophoblast (7)	1.6±1.2	12±17	26±10	0.2±0.1	0.08±0.06 [4]
Fetus (7)	6±5	4±2	33±7	0.3±0.2	0.09±0.05 [4]
Ovary (4)	0.3±0.3	5±8	39±24	0.06 [1]	0.08 [1]
Serosa (uterus) (1)	n. d.	46	21	n. d.	n. d.
Vagina (1)	0.05	2.8	7.1	n. d.	n. d.
Ectocervix (1)	1.6	2.0	9.1	n. d.	n. d.
Endocervix (2)	0.5±0.2	2.7±1.6	46±53	n. d.	n. d.
Uterus (myometrium (6)	0.9±0.6	1.9±0.4	17±4	n. d.	n. d.
Endometrium (3)	0.1±0.1	18±17	13±5	n. d.	n. d.
Fallopian tube (3)	0.8±0.5	1.7±0.4	36±10	0.15±0.1 [2]	0.10±0.02 [2]
Infundibulum (2)	0.4±0.2	16±22	21±5	n. d.	n. d.
Round ligament (1)	0.09	74	21	n. d.	n. d.
Peritoneum (1)	0.38	1.8	15	n. d.	n. d.
Fibroma (uterus) (8)	2±2 [6]	12±18	17±7	n. d.	n. d.

The embryonic tissues come from voluntary interruption of pregnancy, while the adult tissues were taken from patients suffering from menorrhagy or dysmenorrhea. The 27 patients were aged 18–77 years. The results are expressed as means ± SD. The numbers in parentheses indicate the number of patients. The numbers in brackets indicates the number of samples in which the compound was observed, if different from the number of patients.

n.d., not detected

ThTP was consistently found in fetal tissue and in particular placenta, but only sporadically in adult tissues. This also holds, though to a lesser extent, for AThTP. Indeed, among the samples containing ThTP, 53% also contained AThTP, while AThTP was only found in 10% of the samples devoid of ThTP (p = 0.0006). Therefore, as for the samples of Table 2, these data suggest that the presence of AThTP is linked to the presence of ThTP. AThDP was only rarely detected.

We compared the activities of the 25-kDa ThTPase in these tissues (Table 5). The enzyme activity was low in fetus and trophoblast, but it was substantially higher in endometrium and uterus. A high activity was also previously observed in mouse uterus [37] and a high expression of the ThTPase mRNA in human uterus [36], in agreement with our present data. While significant amounts of ThTP are observed in fetal tissues, this compound is below detection limit in adult gynecological tissues (Table 4). These results suggest that 25-kDa ThTPase is important for the regulation of cellular ThTP levels *in vivo*, which has never been demonstrated before.

**Table 5:** Soluble 25-kDa ThTPase activities in human tissues.

	ThTPase activity (nmol.mg^−1^.min^−1^)
Trophoblast	0.03±0.01 a
Fetus	0.09±0.01^b^
Brain (temporal cortex)	0.10±0.04^c^
Endometrium	0.18±0.02
Uterus (myometrium)	0.26±0.01

a , significantly lower than fetus, brain (p<0.05); endometrium, uterus (p<0.001).

b , significantly lower than endometrium (p<0.01) and uterus (p<0.001).

c , significantly lower than endometrium (p<0.01) and uterus (p<0.001).

Mean ± SD, n = 3.

### Thiamine compounds in human semen

Spermatogenesis requires thiamine, as evidenced in transgenic mice lacking the high-affinity thiamine carrier ThTR1 [76]. Null males have hypoplastic testes secondary to apoptosis of pachytene stage spermatocytes, but to our knowledge, no determination of thiamine compounds in human semen has ever been published. Our results show that whole semen contained thiamine, ThMP and ThDP in variable amounts (Table 6). No adenylated thiamine derivatives were found. We observed a positive correlation [*F*(1,10) = 21.36, p<0.0009, R^2^ = 0.68] between ThDP content and number of spermatozoa per mL of semen, which varied from 1.8.10^6^ to 135.10^6^ (azoospermic semen samples were not included in this correlation). No significant correlations were found for thiamine or ThMP. These results suggest that ThDP is mainly localized within spermatozoa. This conclusion is also supported by the observation that ThDP concentration was low in seminal fluid prepared from whole semen. Several studies suggested a relationship between sperm motility and oxidative phosphorylation [77], while other studies suggested that, at least in human sperm cells, glycolysis is a primary energy source for motility [78]. We did not find a significant correlation [*F*(1,10) = 3.890, p = 0.0768, R^2^ = 0.28]) between ThDP content of spermatozoa and the percentage of motility (% of spermatozoa with a progressive motility ≥25 µm/sec at 37°C or ≥20 µm/sec at 20°C), though a tendency seemed to exist. ThDP is a cofactor for transketolase in the pentose phosphate shunt and it was shown that experimental inhibition of the pentose phosphate pathway impaired progressive motility of human spermatozoa [79]. It is therefore plausible that the tendency of highly motile spermatozoa to have a high ThDP content is indicative of the functioning of the pentose phosphate pathway rather than of mitochondrial metabolism. Indeed, the pentose phosphate pathway is the main source for NADPH, required for the reduction of glutathione by glutathione reductase. Reduced glutathione is essential in antioxidant defense for spermatozoa, which have limited antioxidant capacity because of their small cytoplasmic volume. ThDP, a cofactor for transketolase, is essential for maintaining the flux through the oxidative part of the pathway, when the requirement or NADPH is high.

**Table 6:** Thiamine compounds in human semen.

		Thiamine	ThMP	ThDP
		(nmol/L)	(nmol/L)	(nmol/L)
Whole sperm (12)	Controls	30±16	25±14	45±22
	Azoospermia^a^ (1)	60	20.4	6
	Azoospermia^b^ (1)	16	20	21
	Azoospermia^c^ (1)	34	9	<2
	Repermeabilization^d^ (1)	115	2.1	45
Seminal fluids (9)	Controls	30±22	24±19	14±6
	Azoospermia (2)	54±41	12±8	13±9

The samples were from 12 healthy men aged from 25 to 42 years. The abstinence time was 3±1 days and the delay between ejaculation and freezing of the samples was 60±30 min. Seminal fluids were obtained by centrifugation (500× g, 10 min). The results are expressed as mean ± SD. The numbers in parentheses indicate the number of patients. We did not find any significant effects of the delay before freezing on the contents of thiamine derivatives.

a Azoospermia as a result of intake of anabolic steroids.

b Azoospermia as a result of chemotherapy following testicular cancer.

c Azoospermia as a result of vasectomy.

d Repermeabilization after vasectomy (86.10^6^ spermatozoa/mL).

In some samples, a peak eluting close to ThTP was observed. It did however not disappear upon treatment with ThTPase, suggesting that it was not ThTP. Testis and prostate are among the tissues that display the highest expression of 25-kDa ThTPase mRNA [36], [37]. In rat testis, 25-kDa ThTPase mRNA is highly expressed in cells close to the basal lamina of the seminiferous tubules. No signal was observed in mature spermatozoa, probably because the cytoplasmic volume is extremely reduced.

### Thiamine derivatives in the brain

We determined thiamine derivatives in pig, rat, quail and human brain samples (Table 7). ThDP and thus total thiamine levels were much lower in human brain than in rat, quail or pig brain. The C_v_ for ThDP was 5/21 = 0.24, which was the same as the C_v_ for postmortem brains previously obtained [46] and lower than for many other tissues in this study, such as skeletal muscle (0.71, Table 2), heart auricle (0.67, Table 2) or liver (0.64, Table 3). In a previous study, we checked thiamine derivatives in the brain of the baboon *Papio papio* [48] and the mean ThDP content was ∼5 nmol/g of fresh weight. Considering that proteins account for about 13% of fresh weight, this corresponds to 38 pmol/mg of protein, a value twice higher than for human brain, but still much lower than for rodent brain.

**Table 7:** Thiamine derivatives in brain samples.

	(pmol/mg of protein)	Thiamine	ThMP	ThDP	ThTP	AThTP
Pig	Cerebral cortex (3)	13±1	5.5±1.1	66±1	1.5±0.4	n. d.
Quail	Forebrain (3)	0.9±0.1	2.0±0.4	116±21	2.8±0.6	n. d.
Rat	Brainstem (5)	4.3±0.4	10±2	115±12	0.50±0.12*	0.2±0.3
	Right hemisphere (5)	4.4±0.6	9±1	153±65	0.35±0.09	0.1±0.2
	Left hemisphere (5)	5.2±1.6	9±2	148±8	0.36±0.08	0.2±0.3
	Cerebellum (5)	4.4±2.6	14±2	168±54	0.31±0.05	0.1±0.2
Human	Cerebral cortex^a^ (5)	0.2±0.3	3.5±2.6	21±5	0.4±0.3	n. d.

a Peritumoral cerebral tissue, from patients with temporal lobe glioblastoma.

*p<0.05, compared with cerebellum.

The results are expressed as mean ± SD. n.d., not detected.

A previous study on postmortem human brain also did not reveal important regional differences in the distribution of thiamine derivatives, though ThDP levels were somewhat higher than average in mammillary bodies and lower in hippocampus [46].

In human brain, ThTP levels were relatively high, accounting for ∼1% of total thiamine. Even higher ThTP levels were found in quail and pig brain in agreement with previous results [80]. This is mainly due to the presence of very low catalytic activity of the 25-kDa ThTPase in pigs [81], while birds probably have no 25-kDa ThTPase [22]. These results clearly suggest an inverse correlation between brain ThTP content and soluble 25-kDa ThTPase activity.

In rat brain, we compared brainstem, right and left cortical hemispheres as well as cerebellum. There were no important differences between the regions, except that ThTP levels were significantly higher in the brainstem than in the cerebellum (p<0.05). AThTP levels were low and highly variable. While AThTP was found in rat brain, it was always absent in pig, quail and human brain.

### ThTP and AThTP in cultured human and rodent cell lines

Thiamine derivatives were determined in cultured cells from human and rodent origin. We tested human neuroblastoma cells (SK-N-BE), human glioblastoma cells (LN-18), mouse myoblasts (C2C12), mouse fibroblasts (3T3), mouse neuroblastoma (neuro-2a) and rat phaeochromocytoma (PC-12) cells (Table 8).

**Table 8:** ThTP and AThTP in cultured cell lines.

	(pmol/mg of protein)	ThTP	AThTP
**Mouse**	C2C12	0.29±0.02	4.7±0.2
	3T3	n. d.	2.5±0.4
	Neuro-2a	0.9±0.4	0.07±0.09
**Rat**	PC-12	2.2±0.4	2.6±0.5
**Human**	SK-N-BE	0.8±0.3	4.0±1.0
	LN-18	0.7±0.1	20±1

Mean ± SD, n = 3.

n. d., not detected.

Significant amounts of ThTP were detected in all cell lines except 3T3. Surprisingly, AThTP was more abundant than ThTP in all cell lines except the mouse neuroblastoma neuro-2a cells. The highest amount of AThTP was found in human glioblastoma LN-18 cells. It should be reminded that AThTP was also consistently detected in tissues that proliferate quickly (placenta, trophoblast and fetus at an early state of development, Table 4). Therefore the presence of AThTP, ThTP and a low 25-kDa ThTPase activity may be features of undifferentiated fast-growing cells.

### Expression of 25-kDa ThTPase in brain and cell lines from different species

As various phosphatases are able to hydrolyze ThTP, we made several tests to make sure that ThTP hydrolysis observed in extracts from cultured cells is catalyzed by 25-kDa ThTPase. We checked that the pH optimum is around 8.5, characteristic of 25-kDa ThTPase, and that the enzyme activity was observed in the presence of Mg^2+^ and not Ca^2+^ [82]. The substrate concentration used was 10 µM as, at such low concentrations, ThTP hydrolysis by alkaline phosphatase for instance is negligible.

**Figure 2:**
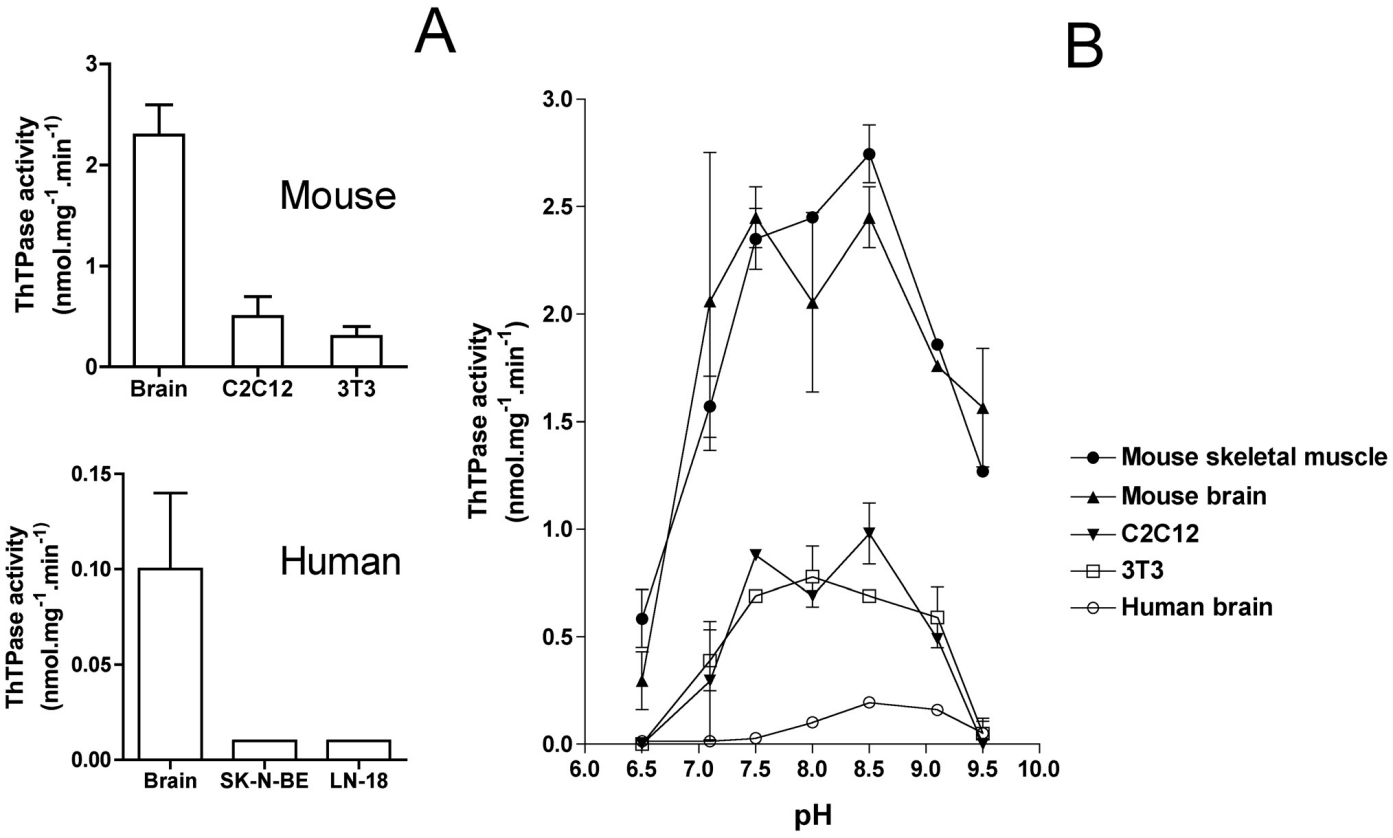
25-kDa ThTPase activities in the brain and cultured cell lines from humans and mice. (A) ThTPase specific activities measured at pH 8.5, at 37°C and at a Mg^2+^ concentration of 10 mM (means ± SD, n = 3) in mouse and human brain and cell lines. (B) pH profile of ThTPase activity from various sources (means ± SD, n = 2). The buffers (50 mM final concentration) used were: Pipes (pH 6.5), Mops (pH 7.1), Hepes (pH 7.5), Taps (pH 8.0), Bis-Tris-Propane (pH 8.5) and Ches (pH 9.1 and 9.5).

25-kDa ThTPase activity is significant in human brain, but ∼20 times lower than in mouse brain (Fig. 2). This might explain the relatively high proportion of ThTP compared to total thiamine in human brain, compared with other species such as rodents. Furthermore, 25-kDa ThTPase mRNA expression was uniformly low in human brain with a somewhat higher expression in the parietal and occipital lobes and a lower expression in the cerebellum [36]. This is also true in mouse brain: both mRNA expression and enzyme activities were somewhat lower in the cerebral compared to the cerebellar cortex [37].

**Figure 3:**
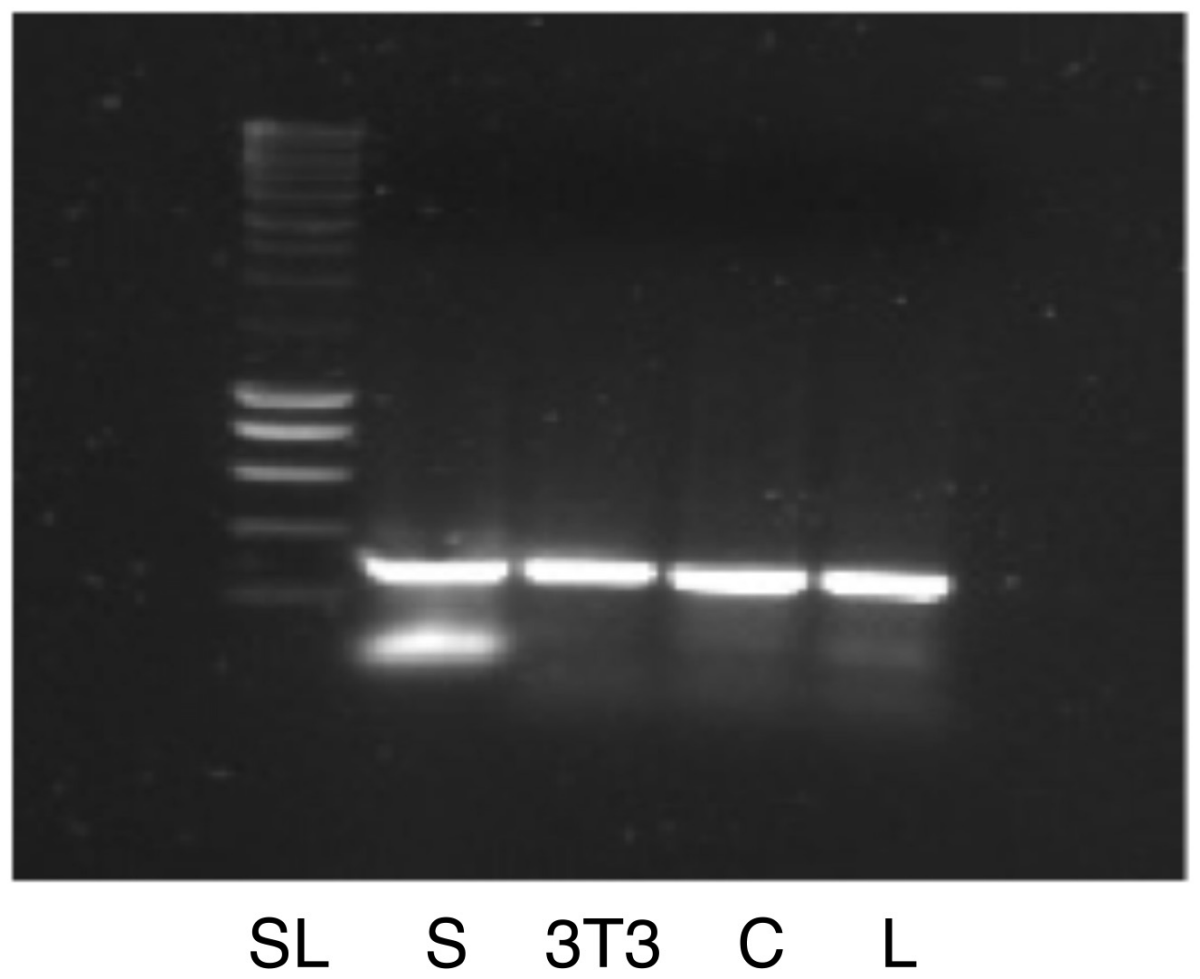
Amplification of 25-kDa ThTPase mRNA in different cell lines by RT-PCR. (SL, standard ladder; S, SN-K-BE; C, C2C12; L, LN-18).

While a significant ThTPase activity could be measured in mouse C2C12 and 3T3 cells, no activity was detected in human SK-N-BE and LN-18 cells. We were unable to measure any significant ThTPase activity in human cell lines. Though the *K*_m_ and *V*_max_ for purified recombinant 25-kDa ThTPase are respectively 175±21 µM and 5.9±0.2 µmol s^−1^ mg^−1^ for the human [82] and a little less than 100 µM and ∼10–12 µmol s^−1^ mg^−1^ for the mouse enzyme [35], these differences do not explain a specific activity in human brain less than 5% of that of mouse brain. Indeed, at the 10 µM substrate concentration used in this study, both enzymes have approximately the same specific activity. Therefore, our data suggest a lower expression of the enzyme in human, compared to mouse tissues. The expression was even lower in cultured cells. This result is surprising as, by RT-PCR, mRNA could be detected in all cell lines (Fig. 3).

**Figure 4:**
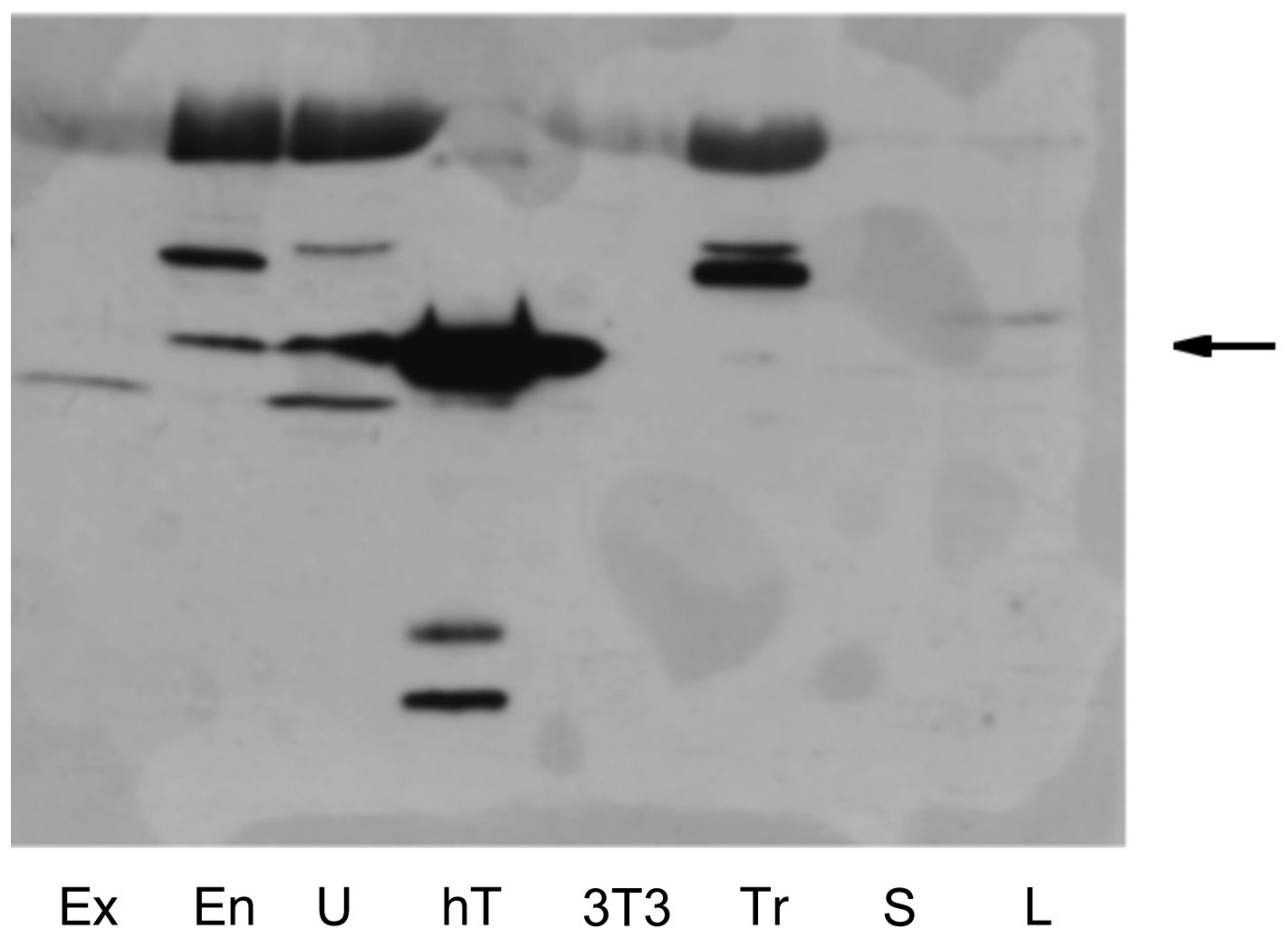
Western blots of human tissues and cultured cell lines using a commercial mouse polyclonal antibody. The antibody was raised against human recombinant 25-kDa hThTPase. The samples were homogenized and centrifuged (20 000× g, 30 min). In the case of the biological samples, 40 µg of cytosolic protein were loaded on the gel; for hThTPase, the amount was 0.5 µg. The arrow indicates the location of 25-kDa hThTPase. (Ex, ectocervix; En, endometrium; hT, human recombinant ThTPase; L, human LN-18 glioblastoma cells; S, human SK-N-BE neuroblastoma cells; U, uterus; Tr, trophoblast).

In order to test whether this is due to the absence of the protein or the presence of a catalytically inactive form of the enzyme, we performed Western blots on human tissues and on SK-N-BE and LN-18 cells using commercial mouse monoclonal and polyclonal antibodies directed against recombinant hThTPase. The monoclonal antibody proved to be useless (see File S1). The polyclonal antibody recognized a band of the correct molecular mass in endometrium and uterus (Fig. 4), but no or possibly only very faint bands of the correct molecular mass were detected in cultured human cells and trophoblast. Purified recombinant 25-kDa human ThTPase [22] was used as a positive control. These results show that, in agreement with enzyme activity determinations, the 25-kDa soluble ThTPase protein is mainly expressed in adult tissues. The presence of mRNA was shown by RT-PCR analysis and therefore we propose that the expression of the protein may be controlled at the level of translation or by posttranslational modification. A similar conclusion has been reached previously for mouse tissues and it was proposed that the highly conserved 200-nucleotide 3′-untranslated mRNA region might be involved in the control of translation [37].

## Discussion

This is the first comprehensive study on the distribution of all known thiamine derivatives in biopsied human tissues and body fluids. Though at least one study reported the distribution of thiamine derivatives in human brain [46], it was a postmortem study and it is known that some derivatives, in particular ThTP, are hydrolyzed during the postmortem delay. No data were available concerning the existence of ThTP and AThTP in human tissues or cultured cells. From the present study we can draw several general conclusions.

### Human tissue ThDP levels are relatively low

Tissue levels of thiamine derivatives have a high inter-patient variability in peripheral tissues. This is not observed in laboratory animals and probably results from external factors, such as nutrition, disease, age, alcohol consumption or drug treatment. Furthermore, it must be emphasized that the biopsies were from patients suffering from various clinical conditions, which might be an additional factor impacting on variability. However, human brain ThDP content, while low, was less variable than in peripheral tissues, suggesting a tight regulation of thiamine homeostasis in brain. This is further emphasized by previous findings that in the thiamine-deficient rat, the rate of disappearance of thiamine is much slower in the brain than in the liver [83], [84].

Total thiamine levels (essentially ThDP, which accounts for most of total thiamine) are much lower in humans than in rodents. Furthermore, human brain total thiamine levels are also much lower than in other animals (Table 7), which could explain the particular sensitivity of the human nervous system to thiamine deficiency. As thiamine levels are already low in human whole blood and plasma, it is possible that intestinal transport of the vitamin is not very efficient in humans. In rodents, the liver contains the highest amount of total thiamine per mg of protein, essentially in the form of ThDP. This, as well as its size, makes it a thiamine store, as recently suggested [85]. This is also underscored by the observation that end-stage liver failure results in thiamine deficiency by depletion of thiamine stores [12]. However, in human liver, total thiamine levels are at least an order of magnitude lower than in rodent liver. Therefore, there is only a very limited storage capacity for thiamine in the human body. This might explain that marginal thiamine deficiency is relatively common in humans. It should be reminded for instance that in the Japanese Navy in the years 1878–1882, each year a third of all enlisted men became sick with beriberi [86]. In rats, overt symptoms of thiamine deficiency occur only after brain thiamine levels drop below 20% of controls, indicating a substantial brain reserve of the vitamin [87]. This threshold value is slightly above the human controls. In one of the rare studies of experimental thiamine depletion in humans, the subjects developed the first clinical symptoms after only two weeks on a diet containing 0.15 mg of thiamine per day [88]. In rodents, complete absence of thiamine in the diet leads to deficiency symptoms only after 4.5 to 9 weeks [87], [89], [90] and generally, the antimetabolite pyrithiamine is administered to accelerate the development of deficiency [91]. These differences between rodents and humans could also, at least in part, be explained by differences in intestinal flora and the presence in the rodent caecum of bacteria able to synthesize thiamine d*e novo*. The fact that thiamine levels might be close to deficiency threshold in humans is further emphasized by the observation that thiamine supplementation can lead to increased well-being [4], [92]. Our results show that, at least in muscle and lung, there is a negative correlation between age and ThDP and total thiamine content. We have previously observed that, in postmortem human brain, ThDP (and total thiamine) levels tended to be highest in very young individuals and decreased in the highest age group (>77 years) [46]. Such a decrease would be in agreement with a decreased thiamine status in elderly people [4], [93], [94], probably as a result of decreased intestinal absorption [95]. Finally, there appear to be many tissue or cell-specific differences in the metabolism of thiamine and thiamine phosphate derivatives, which could explain differences in sensitivity to thiamine deficiency, as already suggested previously for cultured human cells [96].

### ThTP is relatively abundant in human tissues

ThTP is relatively abundant in some tissues, sometimes reaching over 10% of total thiamine (Table 2). This high ThTP content is probably linked to a relatively low specific activity of 25-kDa ThTPase (see below). ThTP is always observed in brain and skeletal muscle. It has been proposed that, in skeletal muscle, ThTP is synthesized by AK1 [25], [26], while in brain it is synthesized in mitochondria by a chemiosmotic mechanism [28]. In the latter case, ThTP can be released from the mitochondria and may be subsequently hydrolyzed by the cytosolic ThTPase.

As for other thiamine derivatives, inter-patient variability was very high for ThTP. In some patients, ThTP was undetectable while in other patients the same tissue contained a relatively high proportion of ThTP. An inverse relationship was observed for thiamine. Therefore, we defined a new parameter, the TPR, reflecting the degree of phosphorylation of thiamine derivatives. This parameter, at least in cardiovascular tissues, seems to be a characteristic of a given patient, at least at a precise moment of his or her life. This parameter tended to be lower in patients suffering from cardiac insufficiency. It is well known that severe thiamine deficiency is often accompanied by acute congestive heart failure. A recent study showed that thiamine deficiency-induced heart failure in the rat involves oxidative stress induced apoptosis [97]. Furthermore, thiamine deficiency impairs contractile function in cardiomyocytes [98]. Though the heart failure is generally attributed to decreased ThDP-dependent enzyme activities, our results suggest that ThTP may also be involved in this phenomenon. Indeed, during thiamine deficiency, both ThDP and ThTP are reduced and our recent results show that ThTP synthesis is probably mostly mitochondrial in the heart [28].

ThTP was rarely observed in gynecological specimen, but was nearly always present in fetal tissue-derived samples. This might be the consequence of a lower 25-kDa ThTPase activity in the latter.

### ThTP levels are probably regulated by cytosolic 25-kDa ThTPase, an enzyme mainly expressed in differentiated cells

ThTP may accumulate in tissues devoid of this enzyme activity, such as *Electrophorus electricus* electric organ [30], pig skeletal muscle [80] or chicken skeletal muscle [26]. Pig and quail brains also have a higher ThTP content than rodent brain (Table 7), which has a high ThTPase activity. The inverse relationship between ThTPase activity and ThTP content also holds for human tissues (Tables 4 and 5): 25-kDa ThTPase activity is lower and ThTP content is higher in fetal tissue and trophoblast than in uterus, for instance. ThTPase activity appeared to correlate with the protein expression as determined by Western blotting (Fig. 4). Strangely, in human cell lines, ThTPase activity, as well as the ThTPase protein, were undetectable. These results suggest that ThTPase expression is linked to the degree of differentiation of the cells. Highly differentiated and quiescent cells such as neurons [38] and, in general, adult tissue in rodents [37] and humans (this study) contain a higher ThTPase activity than non-differentiated fast-growing cells. Though the ThTPase mRNA expression does not correlate with enzyme activities [37], using a semi-quantitative profiling of ThTPase mRNA on a human multiple tissue expression array, we found a low expression in fetal tissue and the highest expression in uterus, testis and prostate, with intermediate expression in liver, skeletal muscle or brain [36]. An increase in 25-kDa ThTPase activity after birth was also observed in rat brain and liver [99].

### AThTP is a minor compound mainly present poorly differentiated fast-growing cells

We show for the first time the existence of AThTP and, very sporadically, of AThDP in human tissues. Interestingly, AThTP is found mainly in fetal or fetus-derived tissues and also in cultured cells but rarely in well-differentiated tissues. Moreover, its presence is at least statistically linked to the presence of detectable amounts of ThTP. It is indeed rarely seen in the absence of ThTP, both biopsies and in cultured cells. This is in contrast to *E. coli* cells in which both compounds to not accumulate together. In *E. coli*, AThTP accumulates in response to carbon starvation or collapse of the transmembrane proton gradient [39], [40], but we have no clue as to the regulation of AThTP levels in animal tissues.

### Conclusions

In summary, the present data substantiate the view that thiamine status is particularly low in humans compared to other mammalian species (especially rodents). This justifies the proposal that thiamine supplementation should be envisaged in many pathological states, especially in elderly patients. An interesting new finding is that ThTP is found in many human tissues at a concentration higher than in other mammalian species, and this is likely due to a lower expression of the specific 25-kDa ThTPase. The possible physiological roles of ThTP and ThTPase are a subject of active investigation in our laboratory. We show for the first time the occurrence of adenylated thiamine derivatives in human tissues. AThTP was found sporadically in many samples, but very consistently in fetal and fetal-derived tissues and cultured cells. It might thus play a role in rapidly dividing cells.

## Materials and Methods

### Materials

All solutions were prepared using milli-Q water (Millipore S.A./N.V., Brussels, Belgium) and all the solvents used for chromatography were of HPLC grade (Biosolve, Valkenswaard, The Netherlands). Thiamine, ThMP and ThDP were from Sigma-Aldrich NV/SA (Bornem, Belgium). ThTP and AThTP, which are not commercially available, were synthesized as described [42], [100]. All other chemicals were from Merck (Darmstadt, Germany) or from Sigma-Aldrich and of the highest purity available.

### Ethics statement

The experimental procedures were in accordance with the Declaration of Helsinki and were approved by the ethical committees of the Hospital of the University of Liège and the Citadelle Hospital (Liège). All tissue donors provided written informed consent.

All animal experiments were made in accordance with the directives of the committee for animal care and use of the University of Liège and in accordance with the European Communities Council Directive of November 24, 1986 (86/609/EEC). The protocols were approved by the Committee on the Ethics of Animal Experiments of the University of Liège (# 823 for rats and # 727 for quails). The animals were killed by decapitation and all efforts were made to minimize suffering.

### Cell culture

Mouse neuroblastoma cells (neuro-2a, ATCC n° CCL-131), mouse myoblasts (C2C12, ATCC n° CRL-1772) and mouse fibroblasts (3T3, ATCC n° CRL-1658) were grown at 37°C in a humidified atmosphere of 95% air, 5% CO_2_, in 10 cm Petri dishes (Greiner Bio-One N.V./S.A., Wemmel, Belgium) in 10 mL of Dulbecco's modified Eagle's medium (Invitrogen Life Technologies, Carlsbad, CA, USA) supplemented with fetal bovine serum (10%). Cells were subcultured to a fresh culture dish when growth reached 70–90% confluence, *i.e.* every 2–3 days. Human neuroblastoma cells (SK-N-BE, ATCC n° RL-2271), human glioblastoma (LN-18, ATCC n° CRL-2610) and rat phaeochromocytoma cells (PC-12, ATCC n° CRL-1721) were grown at 37°C in a humidified atmosphere of 95% air, 5% CO_2_ in 10 cm Petri dishes in 8 mL of DMEM supplemented with 10% heat-inactivated horse serum (Invitrogen) and 5% fetal bovine serum. The medium was changed every 2 days of culture and cells were subcultured to a fresh culture dish when growth reached 70–90% of confluence.

### Preparation of tissue extracts or cultured cells for the determination of thiamine derivatives

Human biopsies were frozen either in liquid nitrogen or on dry ice as rapidly as possible after removal and stored at −80°C until use. Blood samples were analyzed immediately, without freezing. Male Wistar rats (200 g) and adult quails (14 weeks old) were from the institutional animal facility and killed by decapitation. Pig brains were from the local slaughterhouse and immediately frozen on dry ice.

Frozen tissues (approximately 10–50 mg) were homogenized in 10–50 volumes (0.5–1 mL) of trichloroacetic acid (TCA, 12%) in a glass-glass Potter-Elvehjem homogenizer. In some cases, liver samples were homogenized in a medium containing D-mannitol 225 mM), sucrose (75 mM), Hepes (7.5 mM), EDTA (0.5 mM), BSA fatty acid free (0.1%, w/v), pH adjusted to 7.3 with KOH. The homogenate was centrifuged (100 000× g, 60 min) to obtain the cytosolic and the particulate fractions.

Whole semen was precipitated with 2 volumes of TCA (18%). In some cases, fresh semen was centrifuged (500× g, 10 min) to obtain the seminal fluid that was also precipitated with 2 volumes of TCA (18%). We used computer-assisted semen analysis (AutoSperm, MedCalc Software bvba, Mariakerke, Belgium) for the determination of sperm concentration and motility [101]. An aliquot from a homogeneous, mixed semen sample was placed in counting chamber at room temperature and recorded by a video camera. Sperm morphology was assessed using the Krüger strict criteria [102].

Venous blood samples (2×5 mL) were taken from healthy volunteers using EDTA blood collection tubes. The blood was treated with 2 volumes of TCA (18%). For the analysis of human plasma, the blood was centrifuged (1100× g, 15 min, 4°C) and the supernatant treated with 0.2 volumes of 72% TCA.

Cultured cells were detached with trypsin, centrifuged (350× g, 3 min) and the pellet suspended in 500 µL of 72% TCA.

All the samples were centrifuged (5000× g, 5 min), and the TCA was removed by diethyl ether extraction (3×1.5 mL). They were stored frozen at −20°C until determination of thiamine derivatives by HPLC.

### Determination of thiamine derivatives by HPLC

Thiamine compounds were determined by HPLC as previously described after transformation into fluorescent thiochrome derivatives [42], [103]. Prior to analysis, an 80 µl aliquot was oxidized with 50 µl of 4.3 mM potassium ferricyanide in 15% NaOH and a 20-µl sample volume was injected into the chromatographic system (System 522, Kontron Instruments, Milan, Italy). The separation was performed at a flow rate of 0.5 ml/min on a PRP-1 column (Ø 4.1×150 mm, Hamilton Co, Reno, NV, USA) in 50 mM NaH_2_PO_4_ containing 25 mM tetra-*n*-butylammonium hydrogen sulfate and 4% tetrahydrofuran and adjusted to pH 9.5 with NaOH. Thiochrome derivatives were quantified using a fluorometric spectrometer (LS-4, Perkin-Elmer, Shelton, CT, USA, or SFM 25, Kontron Instruments). Proteins were determined in the pellets by the method of Peterson [104] after solubilization in an initial volume of 1 N NaOH.

In some cases, the presence of authentic ThTP was demonstrated by hydrolysis with the specific recombinant mouse 25-kDa ThTPase [35]. The extract (80 µL) was mixed with 15 µL of Bis-Tris-Propane buffer (0.5 M, pH 8.9), 5 µL MgCl_2_ (0.1 M) and 2 µL of the purified recombinant mThTPase (diluted 100×) at 37°C. After incubation (15 min, 37°C), the mixture was put on ice and analyzed by HPLC. In control experiments, the enzyme was replaced by 2 µL H_2_O.

### Preparation of tissue extracts for the determination of 25-kDa ThTPase activity and Western blots

Human tissues were homogenized in a glass-glass Potter-Elvehjem homogenizer with 5 volumes of dilute Tris buffer (5 mM Tris-HCl, 0.5 mM EDTA, pH 8.2) supplemented with protease inhibitors (complete mini EDTA-free Protease Inhibitor Cocktail Tablets, Roche Applied Science, Vilvoorde, Belgium). Homogenates were centrifuged at 4°C for 30 min at 20 000× g. The supernatant was used for the determination of ThTPase and protein concentrations as well as for Western blots.

### RT-PCR detection of 25-kDa ThTPase mRNA in human and mouse cells

Total RNA was extracted from mouse and human cells using RNA InstaPure System (Eurogentec, Belgium) according to the manufacturer's instruction. It was then transcribed in cDNA in 20 µl solution containing 5 µg of total RNA (Promega Benelux b.v., Leiden, The Netherlands).

ThTPase-specific fragments were amplified using specific forward primers (GCT GCA GGA AGT AGC TAG TT and GCA GGA GGT GGC TAG TTT T) and reverse primers (AGC TGT TCA CTT CTA GCA GG and AGA ACT GGC TGC TTC TAG CA) for human and mouse mRNA sequences. The sequence amplified by these primers ranged from nucleotides 342 to 643 and 345 to 645 on human and mouse coding sequence respectively. The PCR reactions using these primers were composed of 1 µl cDNA, 5 µl of each primer (100 µM) and 0.5 µl (2.5 U) Go Taq DNA polymerase in a final volume of 50 µl. PCR cycling parameters consisted of 5 min at 95°C and 35 cycles of 30 s at 94°C, 30 s at 55°C, and 30 s at 72°C, with a final step of 5 min at 72°C (T3000 Thermocycler, Biometra GmbH, Goettingen, Germany). Amplified products were visualized with ethidium bromide staining after electrophoresis on 1% agarose gel. The sequence of PCR products was confirmed by nucleic acid sequencing (GIGA, University of Liège).

### ThTPase activity measurements

Supernatant fractions from tissue or cell homogenates were used for the determination of ThTPase activity [22]. The rate of ThTP hydrolysis was measured in 50 mM Tris-HCl buffer, pH 8.5 in the presence of 10 mM MgCl_2_ and 10 µM ThTP in a final volume of 100 µl. The reaction was stopped by addition of 0.5 mL of 12% TCA. The samples were centrifuged (5000× g, 5 min) and extracted with diethyl ether. The disappearance of ThTP and the production of ThDP were determined by HPLC [103].

### Western blotting

Soluble cytosolic fractions were separated by SDS-PAGE electrophoresis using 0.75 mm thick 12% acrylamide gels. 40 µg of proteins were loaded per well and electrophoresis was carried out at room temperature for 1 h using 20 mA current per gel. 0.5 µg of purified human recombinant 25-kDa ThTPase was used as reference [82]. Proteins were transferred to Hybond-P PVDF Membrane (GE Healthcare Europe GmbH, 201831 Diegem, Belgium) using wet transfer at 4°C for 2 h at 50 V. Membranes were treated for 1 h with TBS-T (50 mM Tris-HCl, pH 7.6, 150 mM NaCl, 0.2% Tween 20, pH 7.4) containing 5% dry milk and then incubated overnight at 4°C with mouse anti-ThTPA poly- and monoclonal (clone 3F6) antibodies (Abnova GmbH, 69126 Heidelberg, Germany) diluted 1∶3000 in TBS-T with 5% dry milk. After incubation, the membranes were washed 3 times for 5 min in TBS-T and incubated at room temperature for 1 h with peroxidase-conjugated AffiniPure F(ab')_2_ fragment rat anti-mouse secondary antibodies diluted 1∶3000 in TBS-T with 5% dry milk. Then the membranes were washed three times in TBS-T and the protein bands were revealed by incubation for 60 s at room temperature using SuperSignal West Pico Chemiluminescent Substrate (Thermo Scientific, Fisher Scientific, Tournai, Belgium).

### Statistical analyses

ANOVA and linear regression analysis were made using Prism 4 or InStat 3 for Macintosh (GraphPad Software). In some cases post hoc comparisons were made using Tukey's test. Coefficients of variation (C_v_) were calculated as the ratios of the standard deviation (SD) to the mean, C_v_  =  SD/Mean. Contingency tables were analyzed using Fisher's exact test.

## Supporting Information

File S1 Western blots of human tissue and cultured cell lines using a commercial mouse monoclonal antibody.
(0.28 MB DOC)
