# Supplementary material for: Thiamine Status in Humans and Content of Phosphorylated Thiamine Derivatives in Biopsies and Cultured Cells
Source: PLoS One. 2010 Oct 25;5(10):e13616. doi: 10.1371/journal.pone.0013616 (PMC2963613; doi:10.1371/journal.pone.0013616)
Supplement: File S1 — Western blots of human tissue and cultured cell lines using a commercial mouse monoclonal antibody. (0.28 MB DOC) [file pone.0013616.s001.doc]

**File S1**

**Western blots of human tissue and cultured cell lines using a commercial mouse monoclonal antibody**

We performed Western blots on human tissues and on human SK-N-BE and LN-18 cells, as well as on mouse 3T3 fibroblasts using a commercial mouse monoclonal antibody directed against recombinant hThTPase (clone 3F6, Abnova GmbH, 69126 Heidelberg, Germany). We used purified recombinant hThTPase [1] as a positive control. In human tissues, the monoclonal antibody recognized a band migrating at a somewhat lower molecular mass, and no band was detected in cultured cells (Figure). Furthermore, if the band detected were 25-kDa ThTPase, this would mean that it would represent approximately 1% (~ 0.5/40, as the signals of this band and of hThTPase are more or less the same) of cytosolic protein content. This is very unlikely as 25-kDa ThTPase is expressed only at a very low level: its mRNA exists only in a few copies per cell [2] and the protein represents only 0.002% of the cytosolic proteins of the brain [3].


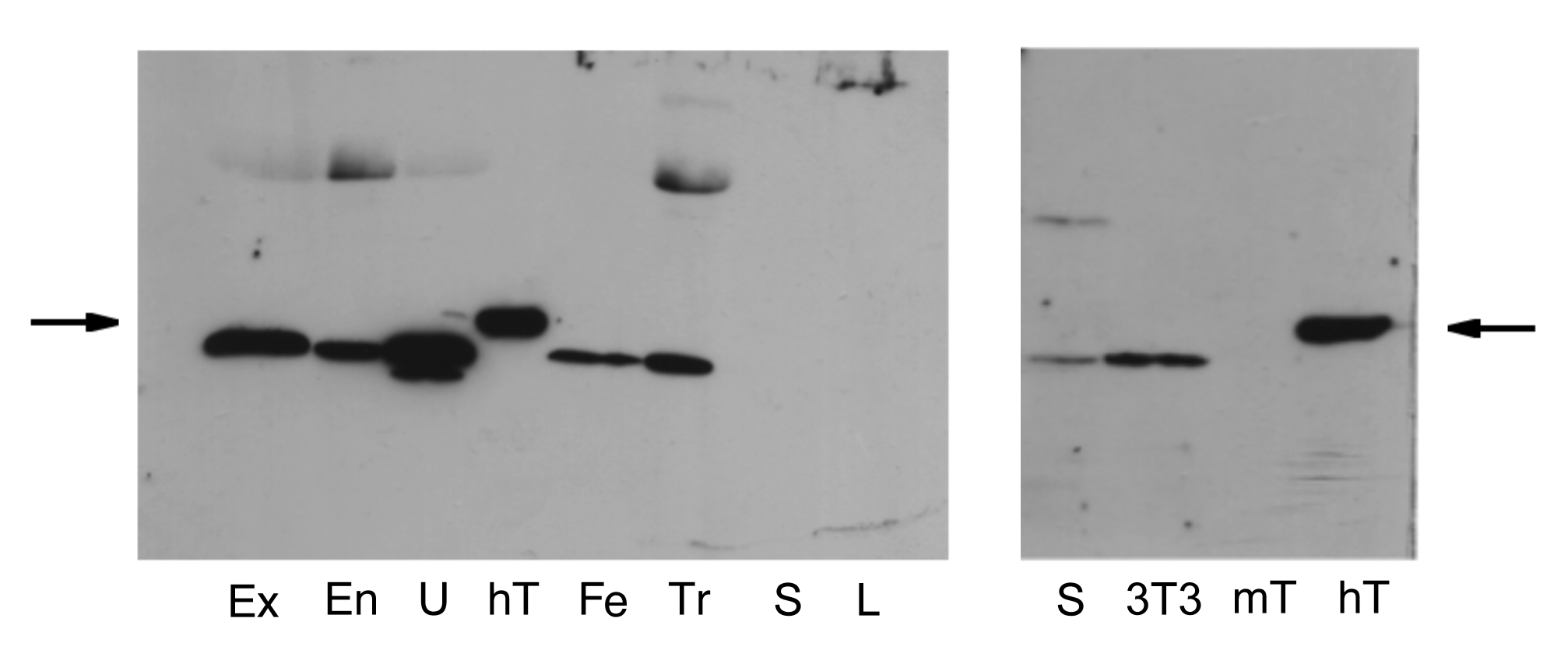


**Figure - Western blots of human tissues and cultured cell lines using a commercial mouse monoclonal antibody, both raised against human 25-kDa hThTPase**

The samples were homogenized and centrifuged (20 000 x g, 30 min). In the case of the biological samples, 40 µg of cytosolic protein were loaded on the gel; for mThTPase and hThTPase, the amount was 0.5 µg. The arrows indicate the location of 25-kDa hThTPase.

(Ex, ectocervix; En, endometrium; Fe, fetus; hT, human recombinant ThTPase;

L, human LN-18 glioblastoma cells; mT, mouse recombinant ThTPase; S, human SK-N-BE neuroblastoma cells; U, uterus; Tr, trophoblast)

Furthermore, the monoclonal antibody did not recognize purified recombinant mouse ThTPase, though it recognized the lower molecular mass band in mouse 3T3 fibroblasts, proving that this band is unrelated to 25-kDa ThTPase. These results show that the commercial monoclonal antibody is useless for laboratory use.

1. Makarchikov AF, Lakaye B, Gulyai IE, Czerniecki J, Coumans B, Wins P, Grisar T, Bettendorff L: **Thiamine triphosphate and thiamine triphosphatase activities: from bacteria to mammals**. *Cell Mol Life Sci* 2003, **60**:1477-1488.

2. Lakaye B, Verlaet M, Dubail J, Czerniecki J, Bontems S, Makarchikov AF, Wins P, Piette J, Grisar T, Bettendorff L: **Expression of 25 kDa thiamine triphosphatase in rodent tissues using quantitative PCR and characterization of its mRNA**. *Int J Biochem Cell Biol* 2004, **36**:2032-2041.

3. Lakaye B, Makarchikov AF, Antunes AF, Zorzi W, Coumans B, De Pauw E, Wins P, Grisar T, Bettendorff L: **Molecular characterization of a specific thiamine triphosphatase widely expressed in mammalian tissues**. *J Biol Chem* 2002, **277**:13771-13777.
